# Considering gene therapy to protect from X‐linked deafness DFNX2 and associated neurodevelopmental disorders

**DOI:** 10.1002/ibra.12068

**Published:** 2022-09-27

**Authors:** Jean Defourny

**Affiliations:** ^1^ GIGA‐Neurosciences, Unit of Cell and Tissue Biology University of Liège, C.H.U. B36 Liège Belgium

**Keywords:** DFNX2, endocochlear potential, gene therapy, hearing loss, POU3F4/Pou3f4, spiral ligament

## Abstract

Mutations and deletions in the gene or upstream of the gene encoding the POU3F4 transcription factor cause X‐linked progressive deafness DFNX2 and additional neurodevelopmental disorders in humans. Hearing loss can be purely sensorineural or mixed, that is, with both conductive and sensorineural components. Affected males show anatomical abnormalities of the inner ear, which are jointly defined as incomplete partition type III. Current approaches to improve hearing and speech skills of DFNX2 patients do not seem to be fully effective. Owing to inner ear malformations, cochlear implantation is surgically difficult and may predispose towards severe complications. Even in cases where implantation is safely performed, hearing and speech outcomes remain highly variable among patients. Mouse models for DFNX2 deafness revealed that sensorineural loss could arise from a dysfunction of spiral ligament fibrocytes in the lateral wall of the cochlea, which leads to reduced endocochlear potential. Highly positive endocochlear potential is critical for sensory hair cell mechanotransduction and hearing. In this context, here, we propose to develop a therapeutic approach in male *Pou3f4* ^−/*y*^ mice based on an adeno‐associated viral (AAV) vector‐mediated gene transfer in cochlear spiral ligament fibrocytes. Among a broad range of AAV vectors, AAV7 was found to show a strong tropism for the spiral ligament. Thus, we suggest that an AAV7‐mediated delivery of *Pou3f4* complementary DNA in the spiral ligament of *Pou3f4* ^−/*y*^ mice could represent an attractive strategy to prevent fibrocyte degeneration and to restore normal cochlear functions and properties, including a positive endocochlear potential, before hearing loss progresses to profound deafness.

## Graphical abstract

Hearing loss is the most frequent congenital sensory disability. About 1–3 in 1000 children are affected at birth or during early childhood by severe hearing losswhich is defined as prelingual deafness, with at least half of all cases having a genetic origin. Mutations and deletions in the gene or upstream of the gene encoding the POU3F4 transcription factor cause X‐linked progressive deafness DFNX2 with inner ear malformations and additional neurodevelopmental disorders. Hearing and speech outcomes after cochlear implantation remain highly variable among patients. *Pou3f4* ^−/*y*^ mice show progressive hearing loss, reduced endocochlear potential, and damaged spiral ligament. Among a broad range of AAV vectors, AAV7 shows strong tropism for the spiral ligament. Thus, we suggest that AAV7‐Pou3f4 transduction in newborn *Pou3f4* ^−/*y*^ mice could help to restore cochlear functions and properties, including a positive endocochlear potential, before hearing loss progresses to profound deafness.

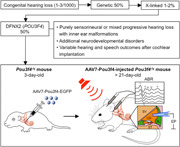

## 1 INTRODUCTION

**Figure 1:**
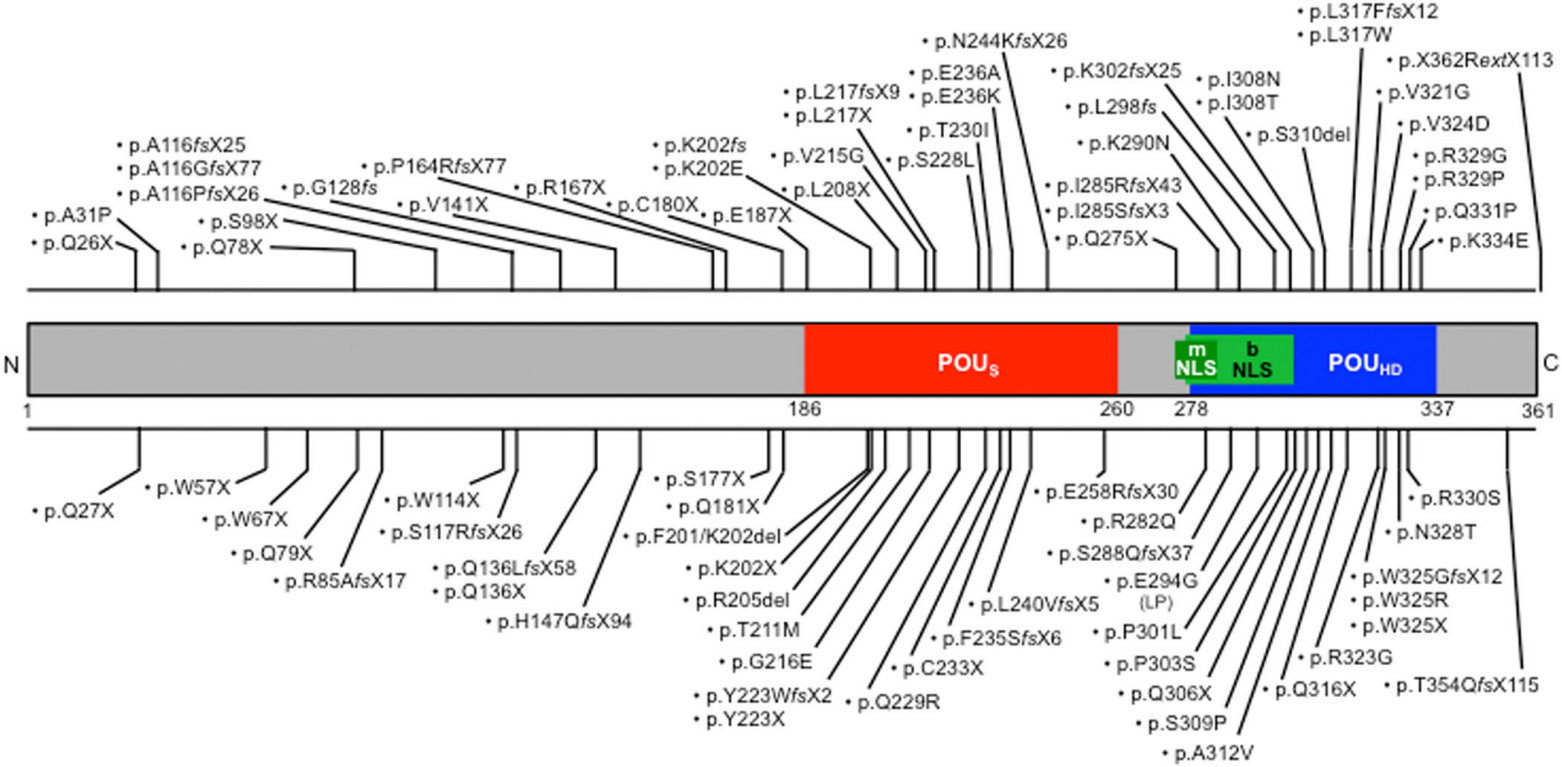
Schematic representation of POU3F4 and localization of pathogenic variants. bNLS, bipartite nuclear localization signal; LP, likely pathogenic; mNLS, monopartite nuclear localization signal; POU_HD_, POU‐homeodomain; POU_S_, POU‐specific domain.

**Figure 2:**
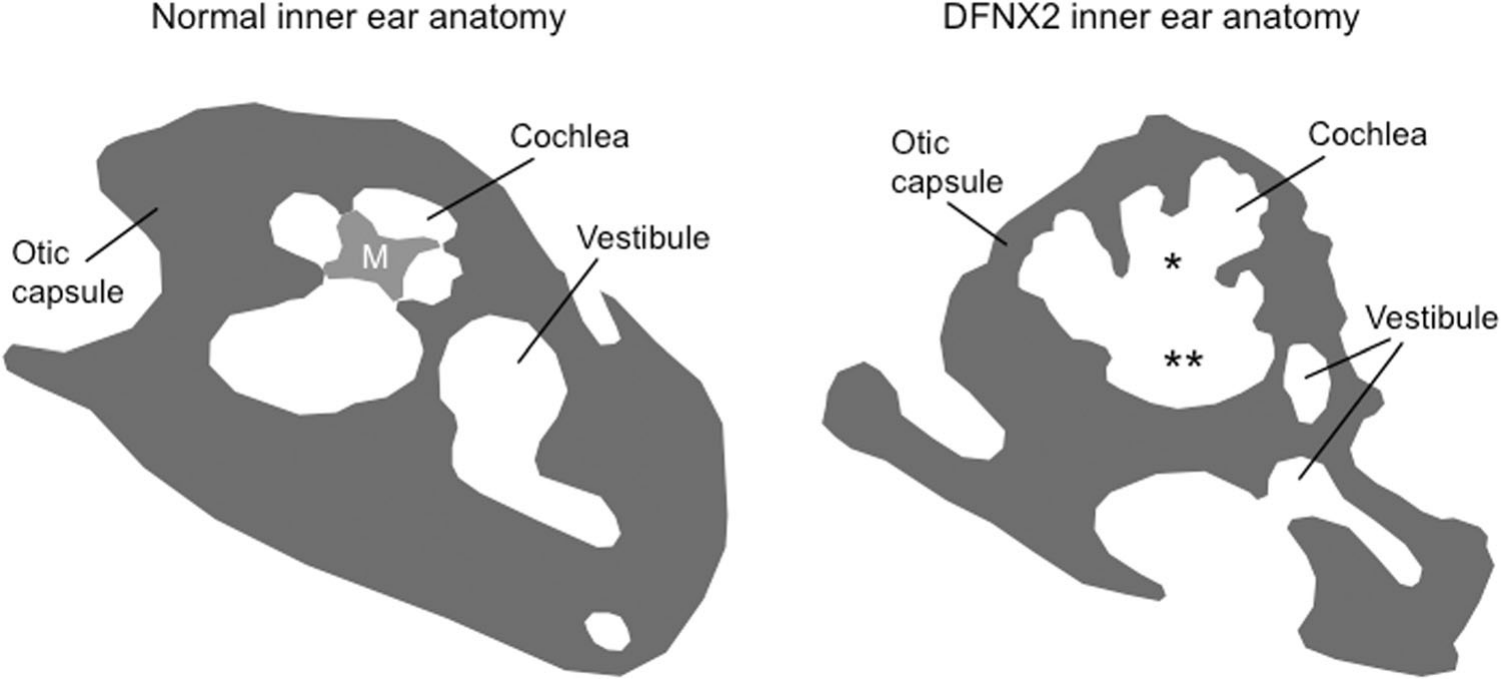
DFNX2 patients develop malformations of the inner ear. Schematic comparison between normal (left panel) and DFNX2 (right panel) inner ear anatomy. In DFNX2 patients, malformations of the vestibule, enlarged internal auditory canal and vestibular aqueduct, cochlear hypoplasia, and absence of modiolus (*) are jointly defined as incomplete partition type III. The lack of bony modiolus results in a fistulous connection between the lateral end of the internal auditory canal and the basal turn of the cochlea (**). The otic capsule (dark gray) appears to be hypomineralized and thinner compared with normal anatomy. M, modiolus.

Congenital deafness is the most prevalent sensory disability. About 1–3 in 1000 children are affected at birth or during early childhood by profound hearing loss, which is defined as prelingual deafness, with 50% of all cases having a genetic origin.^1^ Most of them are inherited in an autosomal recessive manner; however, other types of inheritance also occur, including the X‐linked type related to six loci (DFNX1‐6) and five genes (*PRPS1, POU3F4, SMPX, AIFM1, COL4A6*). About 1%–5% of nonsyndromic hearing loss is likely to be caused by a disease gene on the X chromosome (1/50,000 births).^2^, ^3^ In 1971, Nance et al.^4^ first reported X‐linked mixed deafness with congenital fixation of the stapes and perilymphatic gusher. Mutations in the *POU3F4* gene were first described in 1995 after its localization to the X chromosome in 1988 (Xq21 band).^5^, ^6^ To date, over 80 deafness‐causative mutations in the coding sequence of *POU3F4* have been identified in some 20 countries, including missense, nonsense, deletion, frameshift, and extension mutations (Figure 1).^6^, ^7^, ^8^, ^9^, ^10^, ^11^, ^12^, ^13^, ^14^, ^15^, ^16^, ^17^, ^18^, ^19^, ^20^, ^21^, ^22^, ^23^, ^24^, ^25^, ^26^, ^27^, ^28^, ^29^, ^30^, ^31^, ^32^, ^33^, ^34^, ^35^, ^36^, ^37^, ^38^, ^39^, ^40^, ^41^, ^42^, ^43^, ^44^, ^45^, ^46^ Moreover, deletions of the entire gene as well as deletions, paracentric inversions, and duplications upstream of the gene (containing the putative regulatory elements of *POU3F4* transcription) were also reported.^12^, ^13^, ^24^, ^47^, ^48^, ^49^, ^50^, ^51^, ^52^ X‐linked deafness type 2 (DFNX2, locus Xq21.1), caused by *POU3F4* mutations, accounts for nearly 50% of all cases of X‐linked hearing loss. Although most cases are inherited from the carrier mother, a significant proportion (up to 20% in Eastern Asia) of *POU3F4* mutations also occur de novo.^9^, ^12^, ^17^, ^24^, ^27^, ^33^, ^34^, ^36^, ^40^, ^47^, ^48^ Jang et al.^24^ recently observed a significantly higher de novo occurrence of large genomic deletions within the DFNX2 locus in Korean patients than that of point mutations in the *POU3F4* gene. The relatively high frequency of de novo mutations is one reason for the rather high incidence of sporadic cases of X‐linked deafness DFNX2, meaning that it is not always possible to predict *POU3F4* variants from a family history. Affected males present heterogeneous forms of deafness. Hearing loss can be purely sensorineural or mixed (±50% for both types), that is, with both conductive and sensorineural components, with a variable age of onset and rapid progression to severe hearing loss of all tones in the first decade. DFNX2 patients show anatomical abnormalities on computerized tomography of the temporal bone. Bilateral malformations of the vestibule, enlarged internal auditory canal and vestibular aqueduct, cochlear hypoplasia, and absence of modiolus (i.e., the central bony axis of the cochlea) are jointly defined as incomplete partition type III (Figure 2).^53^ The lack of bony modiolus results in a fistulous connection between the lateral end of the internal auditory canal and the basal turn of the cochlea.^52^, ^53^, ^54^, ^55^, ^56^ A congenital stapedial footplate fixation compromising the ossicular chain mobility in the middle ear is responsible for the conductive component of hearing loss.^4^, ^56^, ^57^ Moreover, Saylisoy et al.^58^ recently suggested that irregular contours of inner ear structures and hypomineralized areas at the otic capsule should be considered as additional criteria for incomplete partition type III. The main inner ear malformations found in DFNX2 patients were recently reviewed by Hong et al.^59^ Female carriers of a *POU3F4* mutation may show no or late‐onset hearing loss.^31^, ^52^, ^60^, ^61^

## 2 *POU3F4* ‐RELATED HEARING LOSS IS ASSOCIATED WITH NEURODEVELOPMENTAL DISORDERS

DFNX2 deafness was originally considered as a nonsyndromic hearing loss.^2^, ^3^ However, further observations of DFNX2 patients revealed additional disorders such as motor and cognitive developmental delays, mental retardation, autism spectrum disorders, learning disabilities, hyperactivity and attention deficit disorders, and oppositional‐provocative behaviors.^9^, ^12^, ^19^, ^33^, ^38^, ^39^, ^42^, ^47^, ^50^, ^52^, ^61^, ^62^, ^63^, ^64^, ^65^, ^66^, ^67^ Recently, an explorative study was carried out focusing on neurodevelopmental symptoms in 10 children with incomplete partition type III. Smeds et al. reported an atypical outcome with poor speech recognition, executive functioning deficits, delayed or impaired language development, and atypical lexical‐semantic and pragmatic skills.^38^, ^67^ Moreover, parents reported mental ill‐health issues with hyperactivity–inattention (restlessness, difficulty concentrating, and a lack of ability to think things out before acting). Overall, these observations suggest that *POU3F4*‐related hearing loss could be considered as part of a neurodevelopmental syndrome that affects the whole child's development as well as hearing. For this reason, Smeds et al.^38^ consider that an extensive and consistent multidisciplinary team approach is required to treat co‐occurring neurodevelopmental disorders during childhood to support overall rehabilitation. In this context, it is worthwhile to mention that a potential association between X‐linked deafness DFNX2 and the presence of hypothalamic malformation, called hamartoma, has been recently reported.^63^, ^68^, ^69^, ^70^, ^71^ Hypothalamic hamartoma is a rare congenital glioneuronal anomaly that can mimic a hypothalamic mass on imaging, without any change in size or spread in the follow‐up. These observations suggest that only about 20% of DFNX2 patients would have normal hypothalamic anatomy.^71^ Clinically, hypothalamic hamartomas are associated with developmental delay, endocrine dysfunction, precocious puberty, gelastic seizures, attention deficit with hyperactivity disorder, conduct and oppositional defiance disorder, rage, and aggression behavior.^72^, ^73^ Over half of children with hypothalamic hamartoma show symptoms of psychiatric comorbidity.^72^ Thus, it is reasonable to assume that some of the neuropsychiatric disorders associated with DFNX2 deafness could have originated from this hypothalamic malformation. However, it should be mentioned that behavioral disorders and hyperactivity could also be a consequence of vestibular deficiency. According to a fairly recent study, the severity of vestibular dysfunction associated with hearing loss is a determinant of comorbid hyperactivity or anxiety.^74^ Vestibular symptoms are common in DFNX2 patients.^8^, ^42^, ^47^, ^75^, ^76^ They have been associated with delayed developmental motor milestones, hypotonia and incoordination in early childhood,^27^, ^38^, ^39^, ^42^, ^47^, ^52^, ^65^, ^66^ and with dystaxia and postural disorders later in life.^8^, ^76^

## 3 CURRENT STRATEGIES TO TREAT HEARING LOSS IN DFNX2 PATIENTS

Current approaches to improve hearing and speech skills of DFNX2 patients do not seem to be fully effective. Stapes surgery (stapedectomy) performed to correct the conductive loss often results in perilymphatic gusher and leakage of cerebrospinal fluid, which can cause dizziness and worsen hearing loss.^4^, ^5^, ^57^, ^77^ Therefore, stapes surgery is contraindicated for DFNX2 patients. In addition, owing to inner ear malformations observed in most DFNX2 patients, cochlear implantation is surgically difficult and may predispose towards severe complications.^78^, ^79^, ^80^, ^81^, ^82^ Because of incomplete separation between the basal turn of the cochlea and the fundus of the internal auditory canal, cochlear implantation may result in electrode insertion into the internal auditory canal without auditory stimulation and risk of facial nerve injury.^78^, ^81^, ^82^ Moreover, even in cases where implantation is safely performed, hearing outcome is highly variable among patients. A recent article reviewed the outcomes after cochlear implantation in patients with DFNX2 deafness.^83^ In most studies, cochlear implantation led to significant improvements in audiometric thresholds and speech recognition compared to preoperative performance.^12^, ^62^, ^82^, ^84^, ^85^, ^86^, ^87^, ^88^ However, several authors report that auditory perception and language development remain globally limited in DFNX2 patients with cochlear implantation.^11^, ^14^, ^22^, ^28^, ^33^, ^38^, ^39^, ^67^, ^89^, ^90^, ^91^ This seems to be particularly true when hearing and speech capabilities are compared with those of cochlear implant recipients without inner ear malformations. In this sense, Smeds et al. reported that very few DFNX2 children with cochlear implantation develop an age‐appropriate expressive language level and are rated to have adequate speech intellegibility.^38^, ^67^ Some studies also highlight the possibility that the benefits of implantation may decline over time (i.e., as the patient grows old).^11^, ^14^ Choi et al.^14^ reported poorer auditory perception scores in DFNX2 patients 2 years after implantation relative to age‐matched cochlear implant recipients without inner ear malformations. Interestingly, this difference was particularly evident in patients harboring large deletions or truncations of *POU3F4*, suggesting that *POU3F4* mutation characteristics may aid in treatment selection and cochlear implantation outcome prediction. Tian et al.^91^ recently reported hearing outcomes in 14 patients with incomplete partition type III and compared them to a control group with normal cochlea anatomy. Auditory thresholds were similar between groups; however, those with inner ear malformation showed poorer consonant recognition 1 year after implantation. In contrast, Alballaa et al.^62^ reported stable audiological outcome 3 years after implantation of patients with incomplete partition type III. Speech recognition scores were lower than average scores for control patients, but without statistical significance.

## 4 *POU3F4*‐DEFICIENT MICE SHOW PROGRESSIVE HEARING LOSS AND REDUCED ENDOCOCHLEAR POTENTIAL

**Figure 3:**
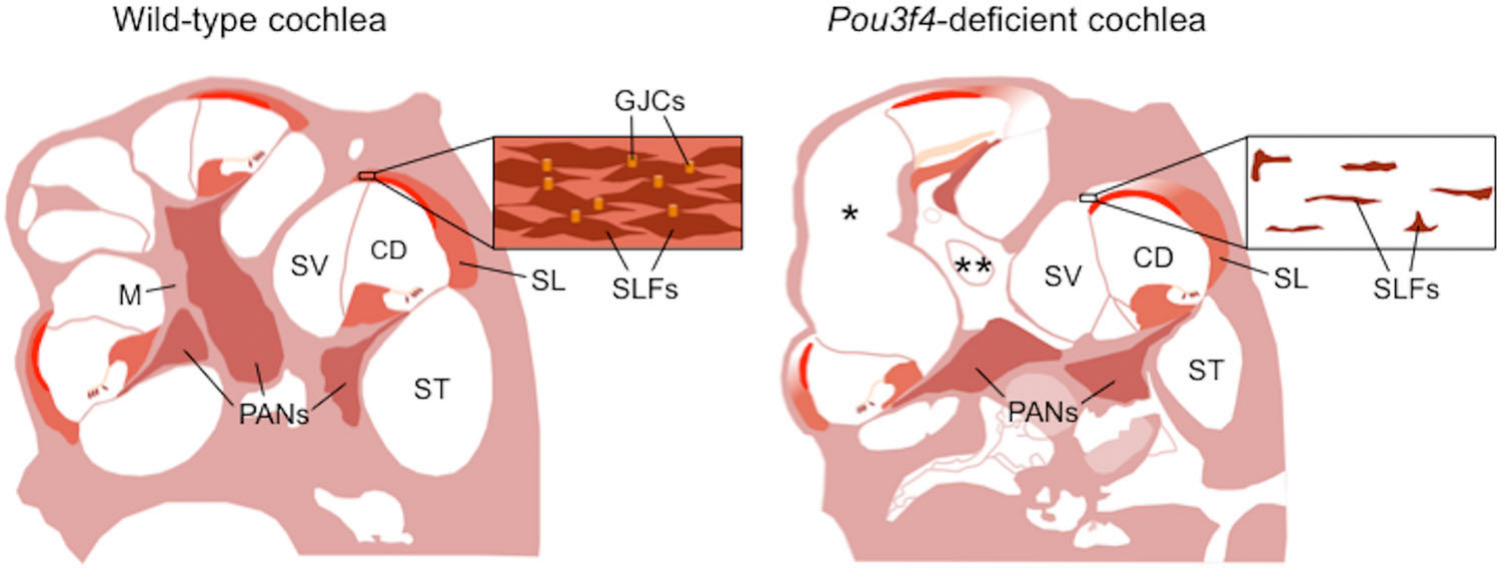
*Pou3f4*‐deficient mice show cochlear hypoplasia and altered spiral ligament fibrocytes. Schematic comparison between wild‐type (left panel) and *Pou3f4*‐deficient (right panel) cochleae. *Pou3f4*‐deficient mice show shorter cochlea (*), absence of modiolus (**), and a severe alteration of spiral ligament fibrocytes (SLFs), especially of those located in the upper portion of the spiral ligament, beyond the stria vascularis (flanking the spiral ligament, in red). In wild‐type mice, SLFs are coupled to each other via gap junction channels (inset in the left panel). In *Pou3f4*‐deficient mice, suprastrial SLFs have a markedly reduced cytoplasmic volume, and the extracellular matrix is extremely sparse (inset in the right panel). CD, cochlear duct; GJCs, gap junction channels; M, modiolus; PANs, primary auditory neurons; SL, spiral ligament; ST, scala tympani; SV, scala vestibuli.

**Figure 4:**
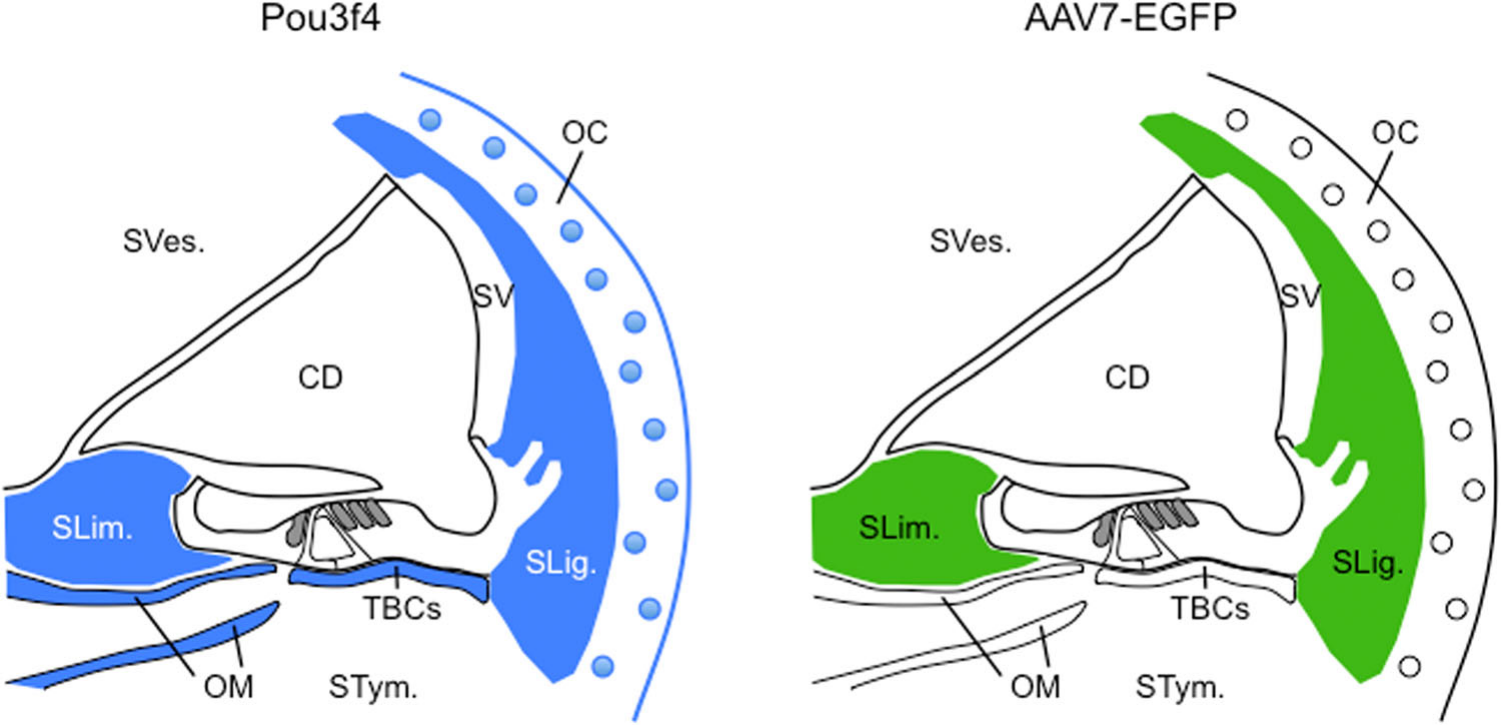
The tropism of the AAV7 vector in the cochlea matches with Pou3f4 expression in the spiral limbus and the spiral ligament. Comparison between Pou3f4 expression (in blue, on the left panel) and AAV7 tropism (in green, on the right panel) in the cochlea. Pou3f4 is mostly expressed in the spiral limbus, otic mesenchyme, tympanic border cells, spiral ligament, and otic capsule. AAV7 mostly transduces the fibrocytes of the spiral limbus and the spiral ligament. CD, cochlear duct; OC, otic capsule; OM, otic mesenchyme; SLig., spiral ligament; SLim., spiral limbus; STym., scala tympani; SV, stria vascularis; SVes., scala vestibuli; TBCs, tympanic border cells.

The first mouse models for X‐linked deafness DFNX2 were generated in the late 1990s.^83^, ^92^, ^93^ At the functional level, loss of Pou3f4 affects middle‐ear sound conduction in mutant animals.^94^ From an anatomical point of view, *Pou3f4*‐deficient mice recapitulate the main inner ear defects observed in DFNX2 patients, such as cochlear hypoplasia and absence of modiolus (Figure 3, right panel), malformations of the temporal bone, the stapes, and the vestibule. These mice show signs of behavioral abnormalities that result from dysfunctions in both the auditory and vestibular systems, including vertical head bobbing, changes in gait, and hearing loss.^93^ Hearing requires the conversion of sound‐induced vibrations into electrochemical signals by mechanosensory hair cells. Sensory transduction in the cochlea depends on fluid movements that deflect the hair bundles located at the apex of mechanosensitive hair cells. Hair cell mechanoreceptors rely on ionic gradients, which allow the passive flow of K^+^ into cells. These electromechanical gradients are achieved by an unusually high K^+^ concentration [K^+^] and a positive potential of the endolymph contained in the cochlear duct, that is, one of the three major fluid spaces of the cochlea, with the adjacent scalae vestibuli and tympani. Both the high [K^+^] (150 mM) and the positive endocochlear potential (EP, +80–90 mV) are generated by the stria vascularis in the lateral wall of the cochlear duct. The ion composition of the endolymph resembles intracellular fluid, whereas that of the perilymph, which is contained in scalae vestibuli and tympani, corresponds to usual extracellular fluids (with ±5 mM [K^+^]). Electrophysiological analyses revealed that *Pou3f4* ^−/y^ male and *Pou3f4* ^−/−^ female mice show progressive hearing loss leading to profound deafness at three months of age, as well as a marked reduction in endocochlear potential.^92^, ^95^ Pou3f4/POU3F4 is a member of the family of POU‐domain (Pit1‐Oct1/2‐unc86) transcription factors with two recognized domains: a POU‐specific domain (POU_S_, between amino acids 186 and 260) and a POU‐homeodomain (POU_HD_, between amino acids 278 and 337), both of which have a helix–turn–helix pattern and determine DNA specificity and binding.^96^, ^97^ POU3F4 was initially predicted to contain three nuclear localization signals (NLS), with one within the POU_S_ and two within the POU_HD_.^29^ However, a recent reanalysis of the sequence using two prediction programs (cNLS Mapper^98^ and NLStradamus^99^) revealed only one monopartite NLS with high confidence between amino acids 275 and 284 (^275^QGRKRKKRTS^284^) within the POU_HD_. If bipartite, the NLS would be most likely located between amino acids 277 and 303. In either case, the POU_HD_, rather than POU_S_, would be critical for nuclear localization (Figure 1). POU superfamily genes are involved in cell proliferation and differentiation during organogenesis.^97^ Little is known about how *POU3F4* mutations induce hearing loss, except that some of them induce subcellular mislocalization of the protein,^15^, ^29^, ^34^ while others lead to the production of a truncated protein,^7^, ^11^, ^13^, ^15^, ^22^, ^23^, ^28^, ^29^, ^40^ or they may affect the structure of the protein and impair DNA binding ability.^7^, ^15^, ^19^, ^29^, ^30^ In other cases, deletions upstream of *POU3F4* that remove noncoding cis‐regulatory elements are likely to affect gene expression.^51^, ^100^ In the cochlea, Pou3f4 is expressed in several structures derived from the otic mesenchyme, including the temporal bone, the spiral ligament, and the spiral limbus. In contrast, no expression was detected neither in the cochlear sensory epithelium nor in the stria vascularis (Figure 4, left panel).^101^, ^102^ Directly flanking the stria vascularis, the fibrocytes of the spiral ligament (SLFs) are believed to ensure the continuous recycling of K^+^ released by the sensory hair cells. A prominent feature of *Pou3f4*‐deficient mice is the severe alteration of SLFs, especially those that are located beyond the stria vascularis (undermentioned “suprastrial”) and that directly border the scala vestibuli. These suprastrial SLFs have a markedly reduced cytoplasmic volume, and the extracellular matrix is extremely sparse (Figure 3, right panel).^92^, ^93^, ^95^, ^103^, ^104^ Such histologic features are reminiscent of a spiral ligament degeneration pattern.^105^ SLF activity is critical for the generation and maintenance of the endocochlear potential.^106^, ^107^, ^108^ Some physiological observations suggest that endolymphatic K^+^ is derived from the perilymph contained in scalae tympani and vestibuli.^109^ In the suprastrial region, SLFs probably resorb K^+^ from the scala vestibuli and then transfer it to the stria vascularis via gap junction channels for return to endolymph by way of Na^+^–K^+^–ATPase activity. Thus, the marked decrease in endocochlear potential measured in *Pou3f4*‐deficient mice as well as the progressive nature of the deafness are likely to be due to the presumed degeneration of suprastrial SLFs.

It is worth noting here that Pou3f4 has been shown to promote axon guidance and survival of primary auditory neurons.^110^, ^111^ As a consequence, *Pou3f4* ^−/y^ mice show reduced afferent innervation of the inner hair cells.^111^ However, given the marked decrease in endocochlear potential, only 30% loss of afferent synapses is likely not a major component of the sensorineural loss in *Pou3f4* ^−/y^ mice. Indeed, a previous study has shown that 50% loss of inner hair cell afferent synapses can occur without affecting hearing thresholds.^112^

## 5 A GENE THERAPY‐BASED APPROACH TO RESCUE THE SENSORINEURAL LOSS IN *POU3F4*‐DEFICIENT MICE

**Figure 5:**
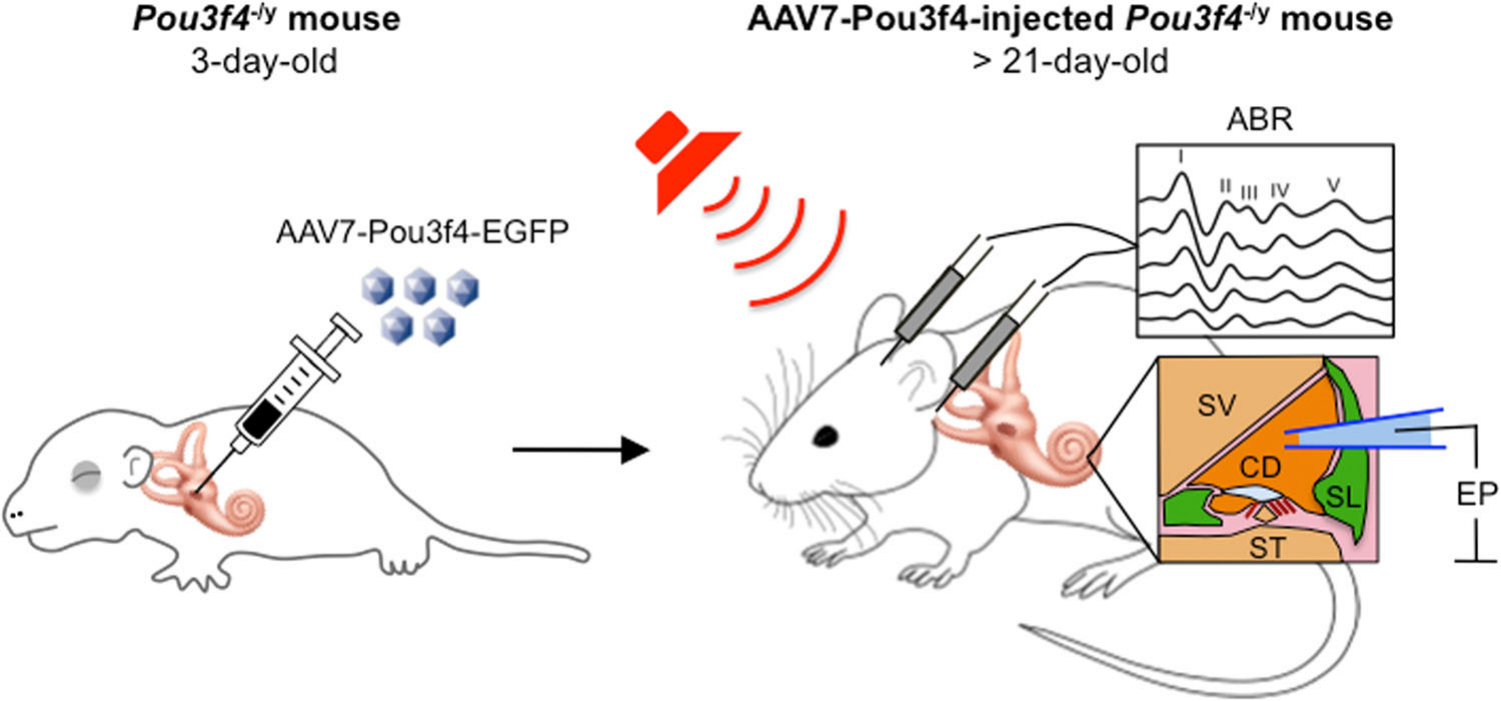
Schematic illustration of an experimental gene therapy protocol for the treatment of Pou3f4‐related hearing loss. Intracochlear viral transduction of the AAV7‐Pou3f4‐EGFP construct should be performed in 3‐day‐old *Pou3f4* ^−/y^ mice (left panel). Auditory brainstem response and endocochlear potential should be measured in AAV7‐Pou3f4‐injected *Pou3f4* ^−/y^ mice from the age of 21 days and the values should be compared with the ones of age‐matched wild‐type and non‐injected *Pou3f4* ^−/y^ mice (right panel). ABR, auditory brainstem response; CD, cochlear duct; EP, endocochlear potential; SL, spiral ligament; ST, scala tympani; SV, scala vestibuli.

As mentioned above, current therapeutic approaches to improve hearing and speech skills of DFNX2 patients still remain a major challenge and these strategies do not seem to be fully effective. In this context, here, we propose to develop a therapeutic approach in male *Pou3f4* ^−/y^ mice based on a viral vector‐mediated gene transfer in cochlear SLFs. Adeno‐associated virus (AAV) is an effective nonpathogenic in vivo gene‐transfer vector that can be used for treating hearing loss in mouse models of human genetic deafness.^113^, ^114^, ^115^, ^116^ Many AAV serotypes have been engineered to improve their transduction to distinct cell types in the cochlea. Very few examples exist of gene therapy experiments to correct inner ear malformations in mouse models of human deafness. A notable exception is the case of Pendred syndrome. Mutations of *SLC26A4*, which encodes pendrin, cause hearing loss associated with enlargement of the vestibular aqueduct. Kim et al.^117^ have shown that a local injection of rAAV2/1‐Slc26a4‐tGFP prevents the abnormal enlargement of the scala media/cochlear duct in *Slc26a4*‐deficient mice. In any case, the viral vector should be chosen to achieve the best match possible with the gene expression profile. Current knowledge suggests that, among a broad range of AAVs, AAV7 appears to be the vector that best matches with Pou3f4 expression in the cochlea. AAV7 especially shows a strong tropism for the entire spiral ligament and for the spiral limbus, whereas this vector minimally transduces the inner and outer sensory hair cells (Figure 4).^118^, ^119^ This point is critical to avoid any potentially deleterious effect of an ectopic expression of Pou3f4. Thus, an AAV7‐mediated delivery of Pou3f4 complementary DNA (cDNA) in the spiral ligament of *Pou3f4* ^−/y^ mice represents an attractive strategy to prevent SLF degeneration and to restore normal cochlear functions before hearing loss progresses to profound deafness. Intracochlear viral transduction of AAV7‐Pou3f4‐EGFP construct should be performed in 3‐day‐old *Pou3f4* ^−/y^ mice, that is, as early as possible and before the presumed degeneration of SLFs begins in these animals (Figure 5, left panel). Owing to malformations affecting the inner ear of *Pou3f4* ^−/y^ mice, different delivery routes could be tested to achieve the best transduction efficiency of cochlear SLFs. The most usual and successful way of delivering vectors or drugs to the inner ear is an intracochlear approach, via the round window membrane. Another attractive option might be to consider direct administration into the scala media compartment via cochleostomy. In the present case, gene therapy is not expected to reverse *Pou3f4*‐related inner ear malformations. Rather, the main objectives should be to examine whether Pou3f4 cDNA transfer in the spiral ligament of *Pou3f4* ^−/y^ mice restores long‐term cochlear functions assessed by auditory brainstem responses and measurement of endocochlear potential. Wild‐type, AAV7‐Pou3f4‐transduced *Pou3f4* ^−/y^, and nontransduced *Pou3f4* ^−/y^ animals should be tested and compared from the age of 21 days (Figure 5, right panel). As mentioned above, cochlear implantation in DFNX2 patients still remains a challenge and may predispose towards postoperative complications. Even in cases where implantation is safely performed, long‐term outcomes of hearing and speech rehabilitation remain uncertain and highly variable among patients. For all these reasons, such a gene therapy protocol could represent an excellent complementary approach to rescue the sensorineural component of the hearing loss. If it works, this original strategy could represent a new hope for improving the quality of life of DFNX2 children and their families. Beyond the potential beneficial effect for DFNX2 patients, this innovative therapeutic approach should represent a major breakthrough that could open up attractive prospects for the treatment of a broad range of SLF pathologies. Several of them have been recently reviewed and discussed by Furness^107^ and Peeleman et al.^108^

## AUTHOR CONTRIBUTIONS

Jean Defourny was involved in the conceptualization of the study and writing of the manuscript—original draft.

## CONFLICT OF INTEREST

The author declares no conflict of interest.

## ETHICS STATEMENT

Not applicable.

## Data Availability

The data that support the findings of this study are available from the corresponding author upon request.
